# Mosquito Oviposition Behavior and Vector Control

**DOI:** 10.3390/insects7040065

**Published:** 2016-11-18

**Authors:** Jonathan F. Day

**Affiliations:** Florida Medical Entomology Laboratory, University of Florida, IFAS, 200 9th St. SE, Vero Beach, FL 32962, USA; jfda@ufl.edu; Tel.: +1-772-778-7200 (ext. 132)

**Keywords:** mosquito oviposition, mosquito control, mosquito-borne disease transmission

## Abstract

The burden of gene transfer from one mosquito generation to the next falls on the female and her eggs. The selection of an oviposition site that guarantees egg and larval survival is a critical step in the reproductive process. The dangers associated with ephemeral aquatic habitats, lengthy droughts, freezing winters, and the absence of larval nutrition makes careful oviposition site selection by a female mosquito extremely important. Mosquito species exhibit a remarkable diversity of oviposition behaviors that ensure eggs are deposited into microenvironments conducive for successful larval development and the emergence of the next mosquito generation. An understanding of mosquito oviposition behavior is necessary for the development of surveillance and control opportunities directed against specific disease vectors. For example, *Aedes aegypti* Linnaeus is the vector of viruses causing important human diseases including yellow fever, dengue, chikungunya, and Zika. The preference of this species to oviposit in natural and artificial containers has facilitated the development of *Ae. aegypti*-specific surveillance and toxic oviposition traps designed to detect and control this important vector species in and around disease foci. A better understanding of the wide diversity of mosquito oviposition behavior will allow the development of new and innovative surveillance and control devices directed against other important mosquito vectors of human and animal disease.

## 1. Introduction

Anautogenous female mosquitoes must blood feed to acquire the protein necessary to develop a batch of eggs. Blood meal digestion is temperature-dependent and takes 2 to 3 days in the tropics and 5 to 8 days in temperate environments [1] (pp. 222–285). Products from blood meal digestion (such as amino acids) are absorbed by the fat body, which in turn synthesizes and releases vitellogenin (Vg), a glycophospolipoprotein, into the hemolymph. The Vg is transported to the ovaries and where it is absorbed by oocytes in the follicular epithelia. Female mosquitoes are considered to be gravid, sometimes containing as many as 250 eggs, when the oocytes are replete. Gravid females initiate oviposition searching flights that correspond with their normal daily activity periods. Many mosquito species oviposit during the dawn and dusk crepuscular periods. Even though mosquitoes have fixed circadian activity periods, environmental factors ultimately control their flight patterns. Meteorological conditions including temperature, rainfall, relative humidity, and wind determine when gravid females fly and where they oviposit. In general, warm, moist, humid, and calm conditions favor mosquito flight and oviposition [2]. Once in flight, gravid females rely on visual [2,3] and olfactory cues to identify potential oviposition sites. As a site is approached, visual, olfactory, and finally tactile cues are used to evaluate the quality of the site for oviposition. The chemical ecology of mosquito oviposition was reviewed by Bentley and Day [4] and is a source of additional information concerning many aspects of mosquito oviposition behavior. It can take a gravid female days, weeks, or even months to locate a suitable oviposition site. Delayed oviposition may affect disease transmission by mosquitoes infected with nematode, protozoan, or viral pathogens. Once ingested, the pathogen undergoes an extrinsic incubation period (EIP) that involves migration out of the blood meal, penetration of the midgut, replication within mosquito cells, and invasion of the mosquito’s body tissues including the salivary glands, a process that may take from 7 to 30 days and is usually temperature-dependent. Because blood digestion is a short process, usually less than a week, it is unlikely that the EIP will be completed before digestion of the blood meal in which the pathogen was acquired. When oviposition is delayed, the EIP may be completed before the first egg batch is laid, thus allowing disease transmission after a single gonotrophic cycle [5].

An understanding of the biological factors associated with mosquito oviposition provides opportunities for the design of highly effective, species-specific mosquito surveillance and control protocols. For example, the preference of some *Culex* species to lay eggs in highly organic freshwater pools has been exploited to develop efficient traps (gravid traps) [6] to monitor mosquito populations responsible for the transmission of St. Louis encephalitis and West Nile viruses. Likewise, the preference of *Aedes aegypti* to oviposit in artificial containers was used to develop ovitraps for the surveillance and control of local populations of this mosquito species in Australia [7].

The diversity of mosquito oviposition behavior provides some of the most interesting examples of adaptation in the natural world. Strategies and adaptations including skip oviposition, where females scatter an egg batch between different oviposition sites; morphological adaptation, where a narrowing of the thorax allows females access to oviposition sites through extremely small holes; and egg-brooding, where females guard and protect their eggs, are all factors that ensure mosquito larval development in favorable habitats. The natural history of mosquito oviposition including oviposition site location and evaluation, physiological factors that affect oviposition, circadian oviposition behavior, mosquito egg-hatching strategies, and how an understanding of mosquito oviposition can be used to develop novel mosquito surveillance and control techniques are the subjects of this review.

## 2. The Natural History of Mosquito Oviposition

### 2.1. Oviposition Site Location 

There are presently 3550 mosquito species recognized worldwide that are contained in two subfamilies, 11 tribes, and 112 genera [8]. Mosquitoes deposit two basic types of eggs: Rapid-Hatch (RH) and Delayed-Hatch (DH). Rapid-Hatch eggs are deposited directly into water, on the water surface, or on substrate close to the water and usually hatch within 48 h. The RH eggs are laid individually, in small groups, or in rafts containing up to several hundred eggs. Delayed Hatch eggs are usually deposited singly or in small groups, are drought-resistant, survive for long periods out of the water, hatch soon after being re-flooded, and sometimes enter a photoperiod-induced diapause to survive temperate and arctic winters. Examples of mosquito egg laying behavior and the types of eggs deposited are presented in Table 1.

Based on mosquito flight behavior in Florida, USA, Bidlingmayer [9] grouped species into three broad categories according to flight preference: (1) Field mosquitoes occurred most frequently in habitats such as new fields, open salt marshes, and pine flatwoods; all habitats with little understory vegetation. These species occurred in lowest numbers in densely vegetated habitats. Field mosquitoes avoided trees and shrubs and were taken in large numbers in light traps set in open habitats. Examples of field species include *Aedes taeniorhynchus* (Wiedemann), *Ae. vexans* (Miegen), *Anopheles crucians* Wiedemann, and *Uranotaenia sapphirina* (Osten Sacken); (2) Woodland mosquitoes preferred forested habitats, avoided open areas during dry periods, and were less likely to enter light traps. Examples of woodland mosquitoes include *Culiseta melanura* (Coquillett) and *Psorophora ferox* (Von Humboldt); (3) Edge mosquitoes preferred ecotone habitats and were intermediate in their response to light traps. Edge mosquitoes include *An. quadrimaculatus* Say and *Coquillettidia perturbans* (Walker). Bidlingmayer and Hem [10] later added a forth group, commuter mosquitoes. These species rested in woodland habitats during the day, but foraged in open habitats during the evening crepuscular, nocturnal, and morning crepuscular periods. *Culex nigripalpus* Theobald is an example of a commuter species. Gillies [11] in an earlier publication classified mosquitoes into four groups based on their flight behavior. His first two groups were equivalent to Bidlingmayer’s field and woodland mosquitoes but Gillies included two additional groups; domestic mosquitoes, such as *Ae. aegypti*, and cave mosquitoes including *Cx. pipiens molestus* Forskal.

**Figure 1:**
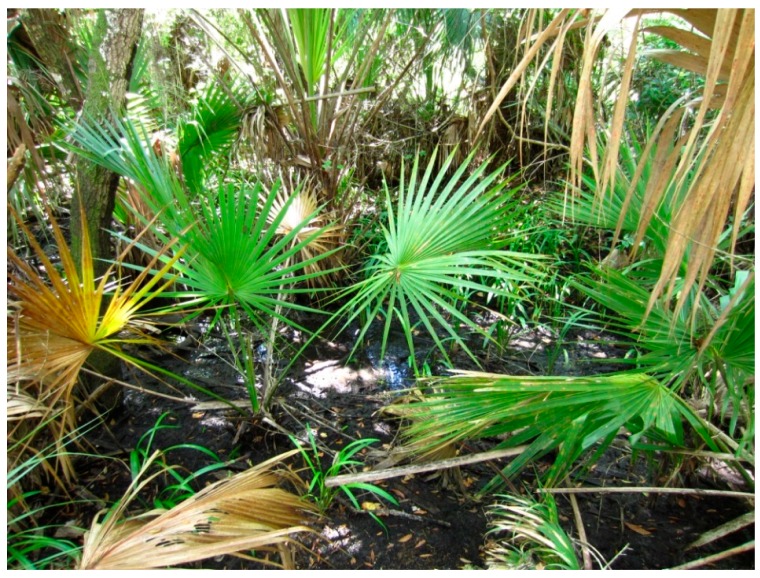
A partially flooded woodland pool in south Florida. This type of habitat is the preferred oviposition site of *Psorophora ferox* which is most abundant during the late summer and autumn.

Habitat preference influences the host seeking and oviposition flights of a female mosquito. Woodland mosquitoes search for hosts in densely vegetated habitats, and following blood feeding and egg development they often oviposit in the same habitat. The ecological, spatial, and temporal distributions of many mosquito species are limited by habitat preference. For example, *Ps. ferox* is a New World woodland mosquito found throughout the eastern half of the U.S., southern Canada, Mexico, Central and South America, and the Caribbean Basin. In subtropical Florida, this species prefers densely-vegetated woodland habitats. Heavy rainfall that floods woodland ground depressions creates temporary shallow pools where this species oviposits and larval development is completed (Figure 1). Rainfall events that flood *Ps. ferox* production sites are commonly associated with autumn tropical depressions, storms, and hurricanes that occur between mid-August and late-October. The dependence of this species on late season rainfall limits the temporal and spatial distribution *Ps. ferox* larvae to autumn woodland pools in south Florida.

The challenges faced by gravid mosquitoes searching for an oviposition site are similar to those faced by their host seeking conspecifics. Gravid and host seeking mosquitoes undertake searching flights as they attempt to locate specific targets. Long- (meters), medium- (meters to centimeters), and short-range (centimeters to contact) cues are used by mosquitoes to identify, approach, and evaluate a target [3]. Many mosquito species are discriminating in their choice of an oviposition site resulting in species-specific larval concentrations and spatial distributions [12]. For example, *Eretmapodites subsimplicipes* Edwards and *Er. quinquevittatus* Theobald are endemic to the coastal forests of Kenya. For *Er. subsimplicipes*, rain-filled fruit husks serve as their primary oviposition site. The husks contain fermented water with strong oviposition attractants and stimulants. Even though the spatial distributions of *Er. subsimplicipes* and *Er. quinquevittatus* overlap, females of the latter species never oviposit in fruit husks. The chemical ecology of fermenting fruit husks provides a mechanism by which closely related sympatric species remain separate and do not compete for limited resources [13].

Much of the oviposition research reported in the scientific literature results from laboratory bioassay studies. While instructive in some ways, laboratory oviposition studies may not accurately reflect the complete natural oviposition sequence. Some of the terminology associated with laboratory bioassay studies has resulted in confusion. Substances are classified broadly as attractants, arrestants, repellents, deterrents, and stimulants. In general, oviposition attractants and repellents tend to act as long- to middle-range cues, causing gravid females to make an oriented movement toward or away from the source [14]. Oviposition stimulants and deterrents act as short-range and contact cues, helping gravid mosquitoes make a final yes-or-no decision relative to oviposition [3]. Stimulants result in egg-laying while deterrents inhibit oviposition at sites where egg-laying would normally occur in the absence of the deterrent. Lowenberger and Rau [15] demonstrated that water containing *Ae. aegypti* larvae parasitized with *Plagiorchis elegans* (Rudolphi) (Trematoda: Plagiorchiidae) received significantly fewer eggs than water containing parasite-free larvae. Even when treatments that held parasitized larvae were sterilized (boiled, treated with antibiotics, or filtered) they continued to receive significantly fewer eggs than treatments holding parasite-free larvae. The authors suggest that a stable oviposition deterrent was present in rearing water that held infected *Ae. aegypti* larvae.

Clarification of the confusing terminology associated with mosquito oviposition will require careful analysis of specific behavioral sequences, similar to studies that have been conducted with other arthropod groups [16]. In most cases, the rigorous experimental studies necessary to quantify and separate attractants from stimulants and repellents from deterrents have not been done. For example, oviposition site selection is often evaluated based on the total number of eggs laid in test versus control sites. While these evaluations accurately indicate the final oviposition site, they say little about the attractants or stimulants that result in oviposition at one site and the repellents and deterrents that prevented oviposition at the other.

The first challenge encountered by gravid mosquitoes seeking an oviposition site in the field is meteorological. Gravid and host seeking mosquitoes require a narrow set of optimum flight conditions. Elevated wind speed reduces the efficiency of most flying insects, including mosquitoes. The normal mosquito flight speed is approximately 1 m/sec. Trap catches, however, are reduced nearly 50% by winds of 0.5 m/s, and by 75% for winds of 1.0 m/s. Trap catches are inversely related to wind speed at all velocities and even the lightest winds reduce mosquito flight [17]. Low temperature, ground moisture, and relative humidity also limit mosquito flight [18]. Light intensity can affect the flight and oviposition behavior of some mosquito species. Many species, including those that are normally nocturnally active, oviposit at twilight and during moon-lit nights [19,20], possibly because the water at oviposition sites reflects light (natural and artificial) rendering them more visible to gravid mosquitoes [21].

Vision is a long-range cue used for oviposition site location and flight orientation by many mosquito species. Visual cues are used to identify and separate aquatic habitats; ponds, streams, bogs, marshes, flooded agricultural land, and natural and artificial containers from parking lots and woodland resting sites [22]. Species that rest in forested areas but oviposit in open habitats, use tree line silhouettes to orient toward woodland areas following oviposition [9]. For domestic species, like *Ae. aegypti*, visual cues likely help them orient in and around buildings and to locate the artificial and natural water-holding containers that they prefer for oviposition [20]. Olfactory cues may also serve as long- mid- and short-range oviposition signals. Aquatic habitats such as freshly flooded roadside and agricultural ditches, agricultural wastewater retention ponds, sewage retention ponds, and wastewater outflow areas contain a mix of organic olfactory signals that are dispersed by the wind.

Once gravid mosquitoes enter areas where potential oviposition sites exist, long-range visual cues again play a role in helping females evaluate and select specific oviposition sites. The water surface itself may present a number of visual signatures. For example, after sunset water holds heat longer than land and the heat is released slowly as infrared radiation. Mosquitoes may be able to visually sense near-infrared radiation (700 to >900 nm) [23] and possibly use it as a long-range oviposition cue. Polarized light [24], ultraviolet light [25], sunlight, and moonlight [21] reflected from the water surface may also be used by gravid mosquitoes to evaluate the location and quality of potential oviposition sites. Gravid mosquitoes have been observed in laboratory and field studies preparing for oviposition while flying over a mirror, and a variety of visual cues must play a role in oviposition behavior prior to actual contact with the substrate [22]. Females of species including *Ae. aegypti*, *Ae. triseriatus* Say, *Toxorhynchites rutilus* (Coquillett), and *Tx. amboinensis* (Doleschall) oviposit in natural and artificial containers and may use visual search images to identify these oviposition sites [26]. Tree-holes and rot holes may present a visual signal that contrasts with the background and invites inspection by gravid mosquitoes.

Mid- and short-range olfactory cues also guide mosquitoes to potential oviposition sites. These cues occur in the form of wind-borne and contact chemicals associated with specific oviposition sites. Sites as diverse as tree-holes, flooded ditches, wastewater retention ponds, cattle hoof prints, and flooded artificial containers produce a range of olfactory signals that in some cases are detected from distances of up to several meters while mosquitoes are still in flight. As mosquitoes approach an oviposition site they use site-specific olfactory cues as short-range signals as they continue to evaluate the quality of the site. Volatile chemical emanations from an oviposition site are sensed and evaluated by olfactory receptors located on the antennae, palps, labrum, and tarsi [27]. Females that oviposit while in flight often make contact with the surface of the water prior to the initiation of oviposition. By doing this they are presumably using chemotactile receptors to evaluate water quality.

### 2.2. Oviposition Site Evaluation

Once a potential oviposition site has been located, the gravid female must evaluate its nutritional quality and probable longevity. When females land on the water, on moist soil adjacent to the water, or on plants prior to oviposition, contact cues help with site evaluation. Surface (water and soil) temperature, texture, and moisture content are probably among the first factors to be evaluated. Contact stimulants in the water are evaluated with antennal, labrum, and tarsal receptors. When females land on soil or vegetation, such as at the edge of a salt marsh or the inside of a tree-hole, substrate texture is evaluated by receptors located on the antennae, labrum, proboscis, tarsi, and abdomen [27,28]. Eggs are sometimes attached to smooth, flat surfaces such as balsam wood, aluminum, and ceramics. However, many mosquito species prefer surfaces that are rough and provide fissures into which the eggs can be deposited. For example, ovitraps containing particle board oviposition paddles with a smooth and rough side are used to monitor *Ae. aegypti* and *Ae. triseriatus* populations. Both species preferentially oviposit on the rough side of the paddle where eggs are sometimes positioned so that only one surface is visible. Five factors associated with oviposition site evaluation by the tree-hole mosquito *Ae. triseriatus* were evaluated in a laboratory study [29] including: (1) the color of oviposition water; (2) the presence of decaying organic matter; (3) the color of the oviposition container; (4) the presence of water in which conspecific larvae were reared to the 4th instar; and (5) the presence of conspecific eggs on the oviposition substrate. Analysis indicated that dark oviposition water and the presence of eggs on the oviposition substrate significantly increased oviposition by this species. Water color and hue are evaluated visually [30] while the presence of eggs on the oviposition substrate is probably evaluated though tactile or chemotactile cues after the female lands.

**Figure 2:**
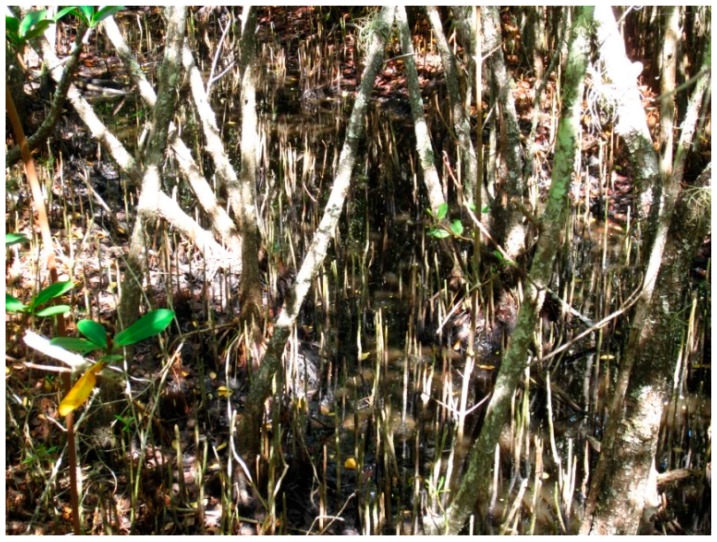
High water mark in a black mangrove (*Avicennia germinans* (Linnaeus) Linnaeus) forest in south Florida where *Aedes taeniorhynchus* females prefer to oviposit in the leaf litter above the standing water.

The moisture content of a potential oviposition substrate is evaluated with moisture-sensitive receptors located on the female’s antennae, labrum, proboscis, tarsi, and abdomen. Salt marsh mosquitoes (*Ae. sollicitans* (Walker) and *Ae. taeniorhynchus*) lay eggs on moist soil above the mean high tide mark where the eggs are periodically inundated by seasonal high tides and fresh water runoff (Figure 2). Eggs deposited in this habitat may not flood for many months, but the eggs are placed onto a substrate that will not be subjected to unreasonable drying and desiccation between flood events. In laboratory studies [31] it was reported that both species normally selected oviposition sites where moisture was equivalent to 70% of the soil saturation moisture content (SMC). The soil SMC is defined as the maximum amount of water held in puddled soil without free water collecting in a depression made in the soil mass [32] (pp. 240–241). Soil SMCs as low as 17% supported the embryogenesis of mosquito eggs. Extremely low soil SMCs were, however, prone to drying and eggs laid under these conditions did not survive. *Psorophora columbiae* (Dyar and Knab) females oviposit on moist soil in flood-prone pastures and agricultural habitats. Laboratory and field studies [33,34] indicate that this species selects oviposition sites where the moisture content ranges from 75% of the soil SMC to complete soil saturation.

When a female mosquito makes contact with the substrate prior to oviposition, stimulants contained in the substrate are important for site evaluation [4]. Oviposition stimulants originate from at least 4 sources: (1) the site itself; or stimulants that result from the presence of (2) mosquito eggs; (3) larvae; and/or (4) pupae. The site itself produces a variety of chemical signals. This is especially true for sites like agricultural land, pastures, roadside ditches, floodwater retention ponds, and woodland ponds that dry down and are periodically re-flooded. After drying, vegetation collects in these sites (Figure 1) and, upon re-flooding, the site produces a rich organic infusion that is highly attractive to some mosquito species. Recently flooded habitats are more attractive to gravid females of some mosquito species than are sites that have remained flooded for long periods [5], suggesting that the attraction of recently flooded sites may be of short duration. Different species, however, show diverse preferences for the age of organic infusions. For example, in coastal southeastern Florida, freshly flooded citrus grove furrows provide a major oviposition habitat for at least 4 mosquito species: *Cx. nigripalpus*, *Ae. vexans*, *Psorophora howardii* Coquillett, and *Ps. columbiae*. The organic concentration of the infusion in newly flooded furrows peaks two to four days after flooding. Mosquito oviposition and egg hatching coincides with the peak organic concentration in the furrow habitat. The infusion clears and settles slowly, and within 10 days, if the furrows are still flooded, the remaining water is clear and mosquito larvae are no longer present [35,36].

Some species prefer older infusions. *Culex quinquefasciatus* Say prefers to oviposit in water with a high organic content like that found in dairy and human wastewater holding ponds and runoff from agricultural treatment plants. In laboratory studies, this species preferred infusions that were 2 to 4 weeks old [37]. Constructed wetlands, storm water retention ponds, and wastewater treatment facilities are becoming more prevalent and are required and regulated by many modern building codes. These habitats provide excellent aquatic environments for mosquito oviposition. It is becoming increasingly evident that the design of constructed wetlands is important for mosquito control. Shallow constructed wetlands with dense vegetation produce large mosquito populations while deep, steep sided constructed wetlands are not favored as oviposition sites by gravid mosquitoes. It is important that an understanding of mosquito egg-laying behavior be incorporated into the design of modern constructed wetlands [38].

Fermented Bermuda grass (*Cynodon dactylon* (Linnaeus)) contains a powerful oviposition attractant/stimulant for *Culex* and *Aedes* mosquitoes [39]. At least 10 compounds that are the products of bacterial catabolism and elicit mosquito oviposition activity have been isolated from Bermuda grass infusion including: 3-methylindole (skatole), p-cresol, nonanal, 2-undecanone, 2-tridecanone, naphthalene, dimethyltrisulfide, 4-ethylphenol, indole, and phenol. A blend of all 10 compounds enhanced oviposition over a wide range of concentrations. When compounds were tested individually, only skatole consistently stimulated mosquito oviposition, suggesting that gravid females of some species are attracted to oviposition sites and simulated to oviposit by blends of compounds and not by individual chemicals [40]. In laboratory studies, Trexler et al. [41] evaluated organic infusions of fermented white oak (*Quercus alba*) as an oviposition attractant and stimulant for *Aedes albopictus* (Skuse) and *Ae. triseriatus*. *Aedes albopictus* preferred oviposition treatments that contained infusion, regardless of its concentration or age, to containers of distilled water. The greatest numbers of eggs were laid in treatments containing 7-day-old, 60% infusion water. *Aedes triseriatus* preferred to oviposit in treatments that fermented for at least 30 days. Binary sticky screen bioassays that capture gravid females before they are able to touch the substrate indicated no difference between the numbers of gravid females attracted to oak infusion vs. distilled water. This suggests that the oviposition response of these species is initiated by a contact stimulant contained in the water rather than by a volatile olfactory attractant.

In some cases, gravid females use the presence or absence of mosquito eggs to evaluate a potential oviposition site. Some eggs are deposited with a volatile aggregation pheromone contained in an apical droplet. For example, *Cx. quinquefasciatus* females produce an apical droplet that accompanies each egg as it is deposited. The major pheromone component in *Cx. quinquefasciatus* apical droplets is (−)-(5R,6S)-6-acetoxy-5-hexadecanolide. This substance induces gravid females to oviposit around the pheromone source. The pheromone was successfully synthesized in the laboratory [42] where it was observed that a shortening of the alkyl chain made it less attractive while perfluorination of the alkyl chain or substitution of the acetoxy groups with the trifluoroacetoxy group resulted in active pheromone analogues. Once the pheromone was synthesized it could be manufactured in large quantities. It was formulated into tablets that were field tested in Kenya for their ability to concentrate gravid mosquitoes into specific production sites where larvae were successfully controlled. The effect of *Culex* apical droplets is nonspecific. For example, *Cx. quinquefasciatus* will display a positive oviposition response when exposed to apical droplet pheromones produced by *Cx. pipiens molestus* and *Cx. tarsalis* Coquillett. Similar aggregation pheromones have been reported for a number of mosquitoes that deposit egg rafts including species of *Culex*, *Culiseta*, and *Uranotaenia* [43,44].

In the case of *Aedes* mosquitoes, laboratory studies dealing with the presence of eggs on oviposition substrates sometimes produce contradictory results. Allan and Kline [45] found that significantly more eggs were laid by *Ae. aegypti* females on oviposition strata that contained conspecific or *Ae. albopictus* eggs than on strata containing no eggs. The same was not true for gravid *Ae. albopictus* females that deposited eggs regardless of the presence or absence of mosquito eggs on the oviposition strata. However, field studies conducted in Trinidad [46] indicated that *Ae. aegypti* females avoided ovitrap paddles that contained conspecific eggs. When females did select paddles containing conspecific eggs, significantly more oviposition occurred on paddles that contained fewer than 25 eggs than on paddles containing more than 25 eggs. For species such as *Ae. aegypti*, that oviposit in small to medium sized artificial containers, it appears that an ability to determine the presence of viable conspecific eggs would be helpful. While the presence of some eggs may indicate a productive oviposition site, too many eggs may result in competition for limited larval resources. A female’s ability to evaluate potential larval competition prior to oviposition and her ability to avoid sites that will contain too many larvae may provide her offspring with a competitive advantage. To test this, Edgerly et al. [47] manipulated *Ae. triseriatus* oviposition sites in the field by adding conspecific larvae and eggs. They tested 3 hypotheses: (1) that gravid females avoid supposed larval competition by selecting sites containing low numbers of conspecific immatures; (2) That gravid females use the presence of conspecific immatures to judge oviposition site permanence and productivity; (3) That females scatter their eggs between oviposition sites. Results indicated that females selected oviposition sites containing low numbers of conspecific immatures early in the year. This behavior, however, decreased as the summer progressed and existing eggs entered diapause. Female *Ae. triseriatus* appeared to judge habitat permanence, as well as potential larval competition, when selecting an oviposition site and usually deposited all of their eggs at one site.

In a similar manner, females are faced with a difficult choice when larvae are present at an oviposition site. The presence of larvae may indicate a superior oviposition site, but too many larvae may compete for limited resources with newly hatched larvae. In spite of studies [48] that suggest the apparent larval-produced oviposition pheromones are sometimes the result of bacterial contamination, there is evidence that oviposition cues of larval origin do occur. Mosquitoes that oviposit in rock-pools are particularly likely to use larval and pupal oviposition cues. *Aedes atropalpus* (Coquillett) and *Ae. togoi* (Theobald) prefer to oviposit in temporary rock-pools and there is laboratory evidence that both species prefer to oviposit in larval rearing water [49,50]. The rearing water attractant appears to be a contact stimulant that is stable and can be stored in solution for weeks. It can be reconstituted to its original activity level after the rearing water is evaporated to dryness. This is obviously advantageous to a species that utilizes oviposition sites prone to total drying and rapid re-flooding. Probably the best evidence in support of an oviposition stimulant of larval origin is from a laboratory study [51] where *Ae. atropalpus* females preferred to oviposit in water that held conspecific larvae maintained at a density of 500 larvae/liter and reared in sterile conditions. In addition, sterile distilled water became a preferred *Ae. atropalpus* oviposition site after 4th instar larvae were present for 48 h.

Substances of pupal origin that stimulate mosquito oviposition have been identified for *Ae. aegypti*, *Ae. atropalpus*, *Ae. caspius* (Pallas), *Ae. togoi*, *Cx. salinarius* Coquillett, and *Cx. tarsalis* in laboratory studies. In the case of *Cx. tarsalis* [52], the active substance was species-specific, had low volatility, and was heat stable because boiled emergence water and reconstituted residue from evaporation treatments retained oviposition attractiveness. However, in several cases [53,54] it appears that bacteria associated with the rearing water were partially responsible for the attractiveness of the bioassays. This again points to the importance of understanding the totality of individual oviposition sites and the difficulty of identifying a single source (eggs, larvae, pupae, or the oviposition site itself) that facilitates or deters oviposition.

A gravid female mosquito uses long- and middle-range cues to approach a potential oviposition site, but still may be repelled from that site by short-range cues or deterred by contact chemotactile substances. Lower and higher aliphatic carboxylic acids are repellents that show activity against gravid *Ae. aegypti*, *Cx. tarsalis*, and *Cx. quinquefasciatus* in the laboratory. Nonanoic acid had the strongest repellent effect for these species. Other substances that showed repellent activity included octanoic, decanoic, acetic, propanoic, butanoic, pentanoic, and hexanoic acids. At high concentrations, these acids cause larval mortality. It has been proposed that gravid females can detect oviposition deterrents that may be toxic to larvae and are signaled by these substances to avoid oviposition at chemically-dangerous sites [55].

Extracts from certain aquatic plant species exhibit oviposition repellent and deterrent properties. This may explain the absence of mosquito larvae in habitats where these plants are abundant. For example, 1,8-cineole is a monoterpenoid found in oil extracts from *Hemizonia fitchii* A. Gray, an aquatic plant that is common in northern California. The 1,8-cineole is not larvicidal, but is a highly effective oviposition repellent [56]. Inorganic salts sometimes deter oviposition in the laboratory and field. Mosquito species including *Ae. taeniorhynchus*, *Ae. sollicitans*, *Ae. togoi*, and *An. albimanus* Wiedemann, that oviposit in brackish water habitats, are more tolerant of salt concentrations than are species that normally oviposit in fresh water. Salt has a negligible vapor pressure, so mosquitoes must be using the presence of salt as a contact stimulant or deterrent. Some mosquito species lay eggs in habitats where salt concentrations are lethal to larvae. For example, *Culiseta inornata* (Theobald) oviposits viable eggs in water with salt concentrations up to 0.1 M, but the saline LD_50_ for larval development of this species is 0.01 M. Apparently, these females choose oviposition sites based on optimal egg hatch rather than optimal larval survival [57]. The presence of *Ae. aegypti* larvae infected with *P. elegans* renders the oviposition site more repellent than does larval crowding or starvation [58]. Deterrents, even in very low concentrations, override the presence of attractants. This relationship may help to maintain larval populations at optimal levels. Deterrents in oviposition substrates may also alert gravid females of potential danger at the oviposition site. Predatory fish and amphibians may signal their presence by the chemicals they produce [59]. The oviposition of *An. punctipennis* (Say) was deterred by water conditioned with chemicals from a single bluegill fish (*Lepomis macrochirus* Rafinesque) [35].

**Figure 3:**
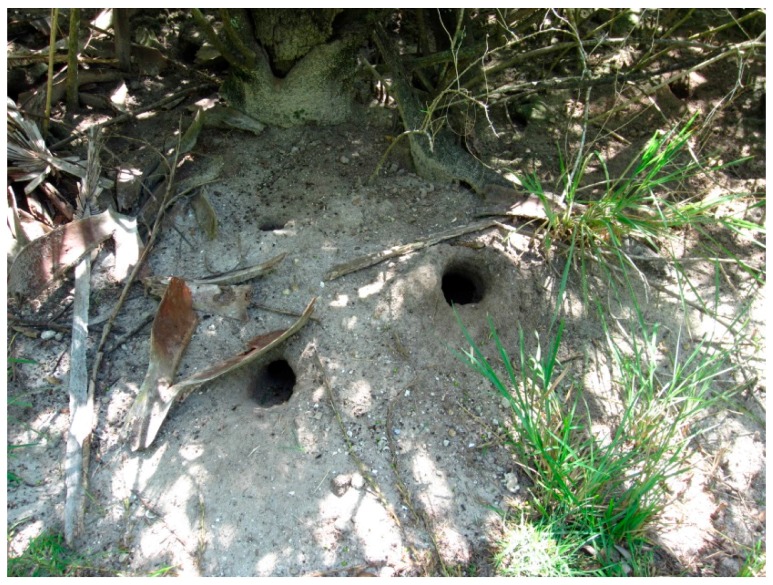
Entrances to three blue land crab burrows in a south Florida salt marsh. The interior of these burrows serves as the major reproductive habitat for the mosquito *Deinocerites cancer*.

The selection of oviposition sites by mosquito species occurs along a continuum ranging from specialist to opportunist. For example, *Ae. atropalpus* and other mosquito species specialize by ovipositing in rock-pools, rock-holes, and coral-holes associated with fast-moving streams, rivers, and ocean-side habitats. *Aedes triseriatus* and *Ae. hendersoni* Cockerell are also oviposition specialists that select tree-holes and artificial containers, such as abandoned tires, as oviposition sites. On the other end of the spectrum are species like *Cx. nigripalpus* that oviposit in virtually any aquatic habitat including salt marshes, tree-holes, plant bracts, birdbaths, other artificial containers, and recently flooded freshwater habitats. Species that oviposit opportunistically, such as *Cx. nigripalpus*, move freely between habitats. Species that are oviposition specialists usually are more restricted in their habitat selection and distribution. Domestic species like *Ae. aegypti*, that oviposit in artificial containers associated with human dwellings generally do not venture far from their oviposition sites. *Deinocerites cancer* Theobald are found in the coastal marshes of southern Florida, the Antilles, Mexico, and Central America where females lay eggs in the partially flooded holes of blue land crabs (*Cardisoma guanhumi* Latreille). Adult *De. cancer* rarely fly far from crab holes where they rest, mate, and oviposit. The spatial distribution of *De. cancer* is closely tied to the environmental and spatial distribution of crabs and their subterranean holes (Figure 3).

In species that share a preference for the same oviposition site, mechanisms have evolved to spatially and temporally segregate them. *Aedes triseriatus* and *Ae. hendersoni* preferentially oviposit in tree-holes. However, *Ae. triseriatus* prefer tree-holes that are close to the ground, while *Ae. hendersoni* prefer those that are high in the canopy, thus avoiding interspecific larval competition. Similarly, of the 4 mosquito species that commonly oviposit in citrus grove irrigation furrows in Florida (see discussion above); *Cx. nigripalpus* females deposit egg rafts as soon as the furrows are flooded. The 3 other species; *Ae. vexans*, *Ps. columbiae*, and *Ps. howardii* lay drought-resistant eggs in moist soil as the furrows dry. *Aedes vexans* females oviposit high in the furrow, usually directly under the tree canopy where hatching is stimulated only by extremely heavy rain (>5 in) or following crown irrigation, when furrows are manually flooded to their tops to prevent tree damage during freezing weather or periods of severe drought. *Psorophora columbiae* and *Ps. howardii* lay eggs low in the furrows where they are hatched by rainfall or irrigation that partially floods the furrows. Partial flooding results in habitat utilization by 3 mosquito species. The larvae of all four species, and maximum interspecific competition, are present only after the furrows are completely flooded. The separation of oviposition sites in the furrows insures that larval competition will be reduced when furrows are marginally flooded [60].

### 2.3. Biotic and Abiotic Factors that Affect the Timing of Oviposition

Many factors may delay mosquito oviposition. Abiotic factors including wind, moon phase, relative humidity, temperature, and the amount of standing water affect the timing of oviposition [61]. Of equal importance are physiological (biotic) factors that influence the ability of gravid mosquitoes to fly, search for an oviposition site, and deposit eggs. One of the most important biotic factors influencing oviposition is the nutritional status of the female. Sugar from nectar, honeydew, or damaged fruit provides mosquitoes with the carbohydrates necessary to generate flight energy. Following adult emergence, most female mosquitoes take at least one sugar meal before blood feeding. Gravid mosquitoes are frequently collected around nectar sources [62]. The nutritional status of a blood fed mosquito is important in determining the ultimate number of eggs that she will develop because sugar feeding increases previtellogenic follicular development in autogenous (the ability to mature eggs without feeding on blood), as well as anautogenous (the requirement of a blood meal for egg development and maturation) species. However, a sugar meal that immediately precedes a blood meal may have the opposite effect by decreasing clutch size. This is because a nectar meal is diverted to the crop and physically occupies space within the abdomen that could otherwise be occupied by blood. Mosquitoes that have ready access to sugar before and after a blood meal may display delayed oviposition, possibly due to reduced flight activity or reduced attraction to oviposition sites [63]. Sugar-starved females, on the other hand, often oviposit indiscriminately. This behavior is frequently observed in the laboratory, where species reluctant to oviposit can be induced to do so through starvation.

Insemination influences the willingness of gravid mosquitoes to oviposit. Shroyer and Sanders [64] reported that both sugar feeding and insemination had an effect on the oviposition of *Ae. vexans* in Indiana, USA. Inseminated females deposited eggs following a single human blood meal sooner and more frequently than did virgin females. It is obviously a selective advantage for virgin females to delay oviposition until they have mated.

Drinking from the surface of an oviposition site is a final pre-oviposition behavior undertaken by many mosquito species. Most females drank more than once and averaged 5 s per drink for a total mean drinking time of 65.7 s [65]. Pre-oviposition drinking is adaptive in several ways. First, it may allow the female one last opportunity to evaluate the quality of the oviposition site. Contact receptors on the tarsi and proboscis are used to evaluate temperature and the presence of stimulants and deterrents. Second, since the average female spends more than 1 min drinking, it is unlikely that this behavior is solely for site evaluation. When females are prevented from drinking by sealing their proboscis, they either fail to oviposit or lay incomplete or abnormally formed egg batches [66]. It appears that pre-oviposition drinking may help the female to properly form and physically expel her clutch of eggs.

### 2.4. Circadian Oviposition Behavior

In a series of papers, Gillett and co-authors [67,68,69,70,71] used the laboratory oviposition behavior of *Ae. aegypti* to help explain the complexities of mosquito circadian rhythm. They reported that oviposition occurred at nearly the same time each afternoon, regardless of ambient temperature. Oviposition, blood feeding, and sugar feeding occurred at about the same time each day as well, and there appeared to be an overall circadian activity cycle that encompassed all of the major behaviors of *Ae. aegypti* females. When mosquitoes were placed in a 12:12 photoperiod in the absence of morning and evening crepuscular periods, eggs were always deposited just before lights-out. When the photoperiod was reversed, the oviposition cycle was also reversed. In fact, mosquitoes could be programmed to oviposit during any time of the day by adjusting the time that the lights were turned off. It didn’t matter whether the day length was extended or shortened; eggs were always laid just before lights-out. If, however, mosquitoes were maintained under conditions of constant light or constant darkness, all cyclical behavior ceased.

By varying the photoperiod it was observed that peak oviposition always occurred 21 to 23 h following the onset of darkness [70]. It appeared that the mosquito’s circadian clock was reset daily by exposure to light. This hypothesis was tested in the laboratory where gravid mosquitoes were maintained under constant darkness and it was observed that oviposition was totally random. After these females received a single exposure to light, ranging from 5 s to 12 h, consistent circadian egg laying cycles began approximately 22 h after return to darkness and continued for 5 days [69]. A similar pattern was not observed when mosquitoes were reared, blood fed, and maintained in constant light followed by a one-time exposure to dark. In a final experiment, mosquitoes were reared and maintained under constant light. Following blood feeding, females displayed asynchronous oviposition. When they were then exposed to constant darkness, an oviposition peak was observed 23 h after the lights were turned off. This 23 h oviposition cycle continued for 5 days after which egg laying again became asynchronous [71].

There is little question that mosquitoes, along with most other plants and animals, have inborn daily rhythms and rely on a balance between light and dark to maintain accurate internal clocks. Females that complete egg development after their normal late afternoon oviposition period retain those eggs until the next afternoon. Under natural conditions, all gravid females in a population experience the same daily photoperiod, so there is a circadian synchronization of oviposition. This is linked with an environmental synchronization in some species, such as *Cx. nigripalpus*, that key on the flooding of oviposition sites following periods of drought as a signal to oviposit [5]. For *Cx. nigripalpus*, their circadian clock tells them when in the 24 h cycle to oviposit and the environmental cue of flooding stimulates ovipositional searching flights. Since photoperiods are seasonal, increasing in the spring and decreasing in the autumn, each individual mosquito’s circadian clock is reset daily and adjusts to seasonal photoperiodic changes.

### 2.5. Mosquito Egg-Laying Behavior

Four egg-laying strategies are observed among mosquito species (Table 1 and discussion above). (1) Some species lay eggs that hatch soon after oviposition (RH eggs), usually within 28 to 36 h. For these species, aquatic habitats must be permanent enough to insure complete larval and pupal development; (2) Some mosquito species deposit desiccation-resistant eggs on moist substrates. These eggs hatch soon after the completion of embryonic development or they dry with the habitat substrate and delay hatching (DH eggs) for periods as long as two years; (3) Some species lay mixed clutches of RH and DH eggs; (4) Some species lay eggs that are protected (brooded) by the female until they hatch. In the case of egg-brooding, the newly deposited batch of eggs is protected from predators and other hazardous environmental conditions such as excessive rainfall.

Species depositing RH eggs are found in many mosquito genera. Relatively few mosquito species deposit groups of eggs in rafts that float on the water surface. Females in the genera *Armigeres*, *Culex*, *Culiseta*, *Coquillettidia*, and *Uranotaenia* lay egg rafts (Table 1). However, egg raft formation does not characterize any of these genera. An interesting alternative to laying egg rafts on the water surface is found in the *Culex* subgenus *Neoculex*, where typical *Culex*-like egg rafts are laid on a substrate above the water line. As the eggs hatch the larvae literally fall into the water. Some species in the genus *Coquillettidia* lay egg rafts that are typically longer than they are wide. In this genus, egg raft shape can sometimes be used for species identification. For example, egg rafts of the African species *Cq*. *fuscopennata* (Theobald) consist of two central rows of about 60 eggs each. The central rows are each flanked by a shorter row containing about 30 eggs. Females from other species, including *Mansonia*, *Ficalbia*, and some *Culex* in the subgenus *Melanoconion* lay clusters of eggs on the surface of the water, on substrate above the waterline, or attached to floating vegetation.

Some species in the genus *Mansonia*, including *Ma. titillans* (Walker) and *Ma. uniformis* (Theobald), lay eggs as underwater clusters attached to leaves, stems, and roots of aquatic vegetation. Some ovipositing females sit on the edge of a partially submerged leaf and extend their abdomen to the underwater portion of the leaf where they lay their eggs. Female *Ma. titillans* oviposit on the undersurfaces of floating water lettuce (*Pistia stratiotes* Linnaeus). At the start of oviposition, females use spines on their 8th tergite to scratch the undersurface of the leaf where the eggs are attached [72]. Embryos in *Culex* and *Coquillettidia* egg rafts are oriented with their heads down so that they emerge directly into the water. *Mansonia* egg clusters that are laid on the underside of submerged vegetation have embryos that are oriented with their heads away from the water surface so that larvae emerge directly into the water. Some members of the genus *Culiseta* lay eggs that hatch soon after oviposition and the oviposition substrate preference of these species varies geographically. Eggs may be laid individually, as rafts on the surface of the water, or as rafts attached to moist substrate 3–5 cm above the waterline.

Most mosquitoes in the genus *Anopheles* deposit individual eggs directly onto the water surface or scatter them across the water as the female hovers above the oviposition site. Females that hover above an oviposition site rely on visual cues to target specific areas for egg deposition [26]. Oviposition site selection may affect the seasonal and ecological distribution of mosquitoes in this genus. For example, Russell and Rao [73] demonstrated that physical cues determine the oviposition site selection and seasonal distribution of the important malaria vector *An. culicifacies* Giles in India. This species lays its eggs in newly planted rice paddies. As rice plants mature and become tall, oviposition slows and eventually stops. By placing regularly spaced vertical glass rods of known height throughout the field, it was demonstrated that *An. culicifacies* females were cuing on the height of rice plants as an indicator of the suitability of paddies for oviposition. When rice plants (and glass rods) reached a height of 30 cm, oviposition ceased. Apparently, mature rice fields provide a less than optimal habitat for *An. culicifacies* larval development and strong selective pressures are in place to ensure that eggs are not deposited in habitats where successful larval development is unlikely.

**Figure 4:**
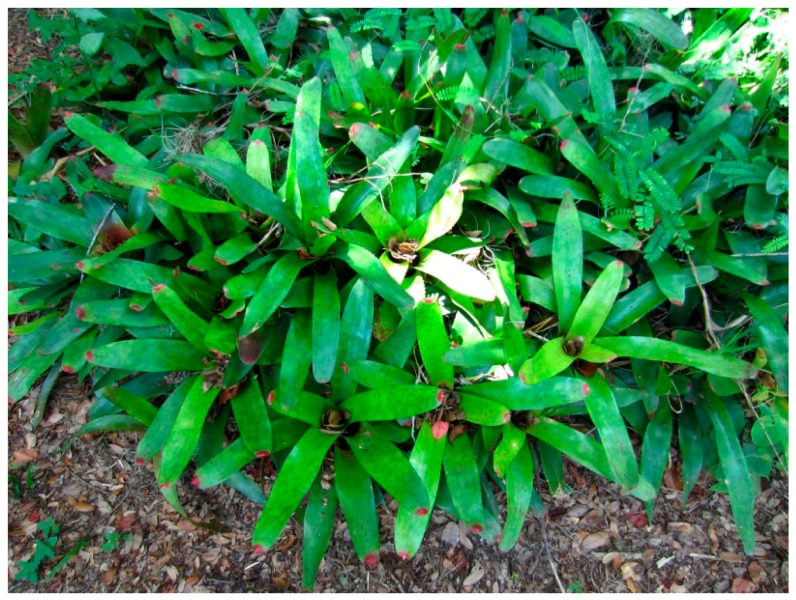
Exotic water-holding bromeliads in south Florida that serve as an oviposition site for *Aedes aegypti* and *Ae. albopictus*. These types of plants are responsible for increased populations of both mosquito species around homes, businesses, and recreational areas throughout Florida.

Water-filled tree-holes, bamboo stems, reeds, or plant bracts often serve as mosquito oviposition sites. When openings to these sites are small, special adaptations are required that allow gravid females to physically reach the water surface or to launch their eggs accurately enough to land in the water. Some species (*Armigeres dolichocephalus* (Leicester) and *Ae. angustus* Edwards) have a greatly narrowed thorax allowing them entrance through small oviposition site openings to lay eggs and the subsequent exit of newly emerged adults from the same oviposition sites. In the case of *Ar*. *dolichocephalus*, oviposition sites are entered through beetle larval holes measuring as small as 1.5 mm in diameter. Female *Sabethes chloropterus* (Von Humboldt) from Central America prefer to oviposit in water-filled bamboo stems. They avoid stems with open tops, preferring instead to shoot their eggs through 1 inch openings in the side of the bamboo stem. Females hover in front of the hole and forcibly eject 1 or 2 eggs at a time through the opening into the water held in the bamboo stem [74]. Members of the genus *Toxorhynchites* oviposit while in flight above oviposition sites that includes tree-holes, bamboo stems, leaf axils, flower bracts, and artificial containers (Figure 4). Gravid females typically fly in counterclockwise ellipses above the oviposition site. After a number of inspection flights (6 to 43 passes), an egg is ejected during the descending portion of an ellipse. During egg ejection the female’s abdomen extends forward through an arc of 80° causing the tip of the abdomen to achieve a speed of ca. 100 cm/s. At this speed, eggs are literally shot from the female’s abdomen and this behavioral sequence allows females to position eggs very accurately onto the water surface [75].

The burden of bridging the generations for mosquito species that lay desiccation-resistant eggs falls to the egg. These eggs are generally deposited away from the water, usually in moist soil and vegetation at the margins of depressions prone to flooding (Figure 3) and above the waterline in partially flooded artificial or natural containers (Figure 4). An embryo’s ability to survive in a drought resistant egg depends mainly on water conservation. Egg chorions are highly permeable to water for the first 2 h following oviposition. Water exchange may continue for up to 16 h, and water loss from newly laid eggs is responsible for desiccation of eggs laid in microhabitats that are too dry. Eggs laid in moist environments actually absorb water and may double in weight due to water gain. Chorionic permeability gradually decreases during the first hours following oviposition as the endochorion tans and eggs darken. After tanning, eggs become more resistant to water exchange. The barrier to water permeability is believed to be a wax layer associated with the serosal cuticle. In some species, the wax is deposited soon after oviposition. For example, the wax layer in *Cx. pipiens* eggs is deposited within 12 min of oviposition. When newly laid eggs of this species are exposed to hydrogen cyanide gas, all embryos die up to the 11 min mark, while eggs older than this survive [76].

Most species in the genera *Aedes*, *Haemagogus*, and *Psorophora* deposit desiccation-resistant eggs. Eggs are deposited individually around the edge of the oviposition site above the high-water mark. The oviposition sites favored by these species are diverse and include woodland pools, agricultural furrows, rock-pools, natural containers including mollusk shells, tree-holes, pineapple tops, and artificial containers including cans, bottles, tires, bird baths, and abandoned boats. Females of some species do not lay all their eggs in one spot. In a study done in Puerto Rico [77], RAPD PCR techniques were used to evaluate the number of families (eggs laid by an individual female) present in oviposition traps. Each female laid an average of 11 eggs. As might be expected, the total number of families rose as the number of eggs laid in each trap increased. However, the total number of eggs laid by each female decreased as the overall number of eggs increased, indicating that females may deposit fewer eggs at oviposition sites already containing conspecific eggs.

Eggs maintained in low humidity desiccate and die, although there is evidence that some eggs can rehydrate when they are returned to high moisture environments. Survival of desiccation-resistant eggs in nature depends on the microhabitat in which they are laid. Even though the overall climate may appear harsh, female mosquitoes of some species are able to select microhabitats that allow egg survival through the coldest winters and the driest summers. *Aedes aegypti* and *Ae. vittatus* (Bigot) eggs deposited in rock-pools survived a Nigerian dry season of more than 4 months where soil temperatures reached 40 °C and relative humidity fell below 5% [78]. Obviously, the microclimate surrounding these eggs was more conducive to survival than the overall macroclimate indicated. Under the proper conditions, desiccation-resistant eggs may remain viable for years, awaiting the proper hatching stimuli that indicate an adequate larval environment is available.

The hatching of mosquito eggs requires flooding or egg deposition in, on, or near standing water as well as completion of embryonic development which is temperature-dependent and species-specific. Embryonic development is usually quick (28–36 h) for RH eggs, such as those laid by *Culex* species, and somewhat slower in DH eggs (see discussion above). For example, the embryonic development of *Ae. aegypti* eggs usually takes 48–72 h, while that of *Ae. africanus* Theobald takes 5–6 days, and it takes up to 11 days for the embryonic development of *Ae. longipalpis* Randolph and O’Neill. Once embryonic development is completed in DH eggs, hatching is postponed until the proper environmental cues and hatching stimuli are present.

Not all mosquito species fall neatly into generalized DH and RH categories. In fact, individuals within a species, especially those that oviposit DH eggs, may show marked variation in where the eggs are laid within an oviposition site and in how quickly eggs hatch after flooding. Egg hatching in some mosquito species has adapted to severe environmental pressures. For example, most anopheline species deposit RH-type eggs; however, some deposit a mixture of RH and DH eggs in temporary habitats, including animal hoof prints and small pools that dry out quickly after flooding. In these habitats, it is advantageous for a female to oviposit both RH and DH eggs. Larvae from RH eggs emerge soon after the completion of embryonic development and take advantage of the flooded habitat. Larvae in DH eggs complete embryonic development, but do not emerge until the habitat re-floods. This is the strategy used by *An. gambiae* in western Kenya, where DH eggs survive in dry soil around temporary oviposition sites. Soil samples collected during the dry seasons of 1987–1989 yielded 126 *An. gambiae* larvae, 2 to 5 days after re-flooding [79]. This RH/DH egg deposition by individual females appears to be a mechanism allowing the maximum utilization of ephemeral oviposition sites. Similar observations have been made for *An. atropos* Dyar and Knab, *An. balabacensis* Baisas, *An. melas* Theobald, and *An. punctimacula* Dyar and Knab. Not all DH eggs at an oviposition site will hatch after flooding, even though they are inundated at the same time and exposed to the same hatching stimuli. The hatching of DH eggs, even those deposited by the same female, are usually staggered and may hatch at irregular intervals over long periods of time. This may be adaptive in preventing a mass emergence into a small temporary larval habitat where food resources are limited.

Some *Mansonia* larvae spend an unusually long time in the egg stage. In the Old World, *Ma. uniformis* and *Ma. africana* (Theobald) larvae spend 6–7 and 7–8 days, respectively, in the egg. In the New World, *Ma. dyari* Belkin, Heinemann, and Page spend an average of 10.5 days in the egg. The ultimate cause of this delayed hatch may result from slow embryogenesis. The delayed hatch response of *Ma. dyari* may be an adaptation to their unusual oviposition strategy. In south Florida, *Ma. dyari* and *Ma. titillans* oviposit selectively on the leaves of water lettuce. Female *Ma. titillans* always oviposit on the underside of floating leaves, while *Ma. dyari* oviposit on both the upper leaf surface, out of the water but near the wet-dry stain line, and on the lower leaf surface under water. During mid-summer, water lettuce plants form dense mats with many of the leaves positioned at a steep angle out of the water. The delayed hatch of *Ma. dyari* may facilitate oviposition on the upper surface of the leaf. By the time delayed embryonic development is complete, water lettuce leaves drop and become submerged, allowing larvae to hatch directly into the water. An added adaptation may be the larvae’s ability to survive for up to an additional 72 h while still in the eggshell. Predator avoidance may provide an additional explanation for oviposition on the upper surface of water lettuce leaves by *Ma. dyari*. *Hyrophilid* and *Dytiscid* larvae are associated with water lettuce plants in nature and members of both groups consumed submerged *Mansonia* eggs in laboratory tests [80].

Perhaps the ultimate oviposition strategy is egg-brooding; the protection of an egg mass by the female that deposited it. Female *Armigeres flavus* (Leicester), along with several other members of the genus, protect eggs that are attached to their hind tarsi. These eggs hatch rapidly when the female immerses her legs and the eggs into water, leading to speculation that this form of egg-brooding protects eggs against parasitism and predation [81]. *Trichoprosopon digitatum* (Rondani) females remain with their egg rafts following oviposition until larvae emerge after about 30 h. Egg rafts are held between the female’s legs as she sits on the water surface. When disturbed, females fly off, but return and appear to search for their egg rafts. When individual eggs are separate from rafts, females appear to try and regroup the eggs [82]. Field and laboratory observations indicate that the brooding females are protecting their egg rafts from being washed out of their exposed cocoa husk oviposition sites by rain [83].

## 3. Mosquito Oviposition for Vector Surveillance and Control

### 3.1. Oviposition Behavior and Vector Surveillance

Two trap types, gravid traps and oviposition traps, have been designed to exploit mosquito oviposition behavior and monitor vector populations. The original gravid trap was developed by Reiter in 1983 [6] as a modified suction trap positioned above a water source that contained an organic oviposition attractant such as 7-day-old hay infusion or an alfalfa infusion made from rabbit chow. Traps that contained organic attractants capture significantly more adult mosquitoes than did traps containing distilled water. Gravid females attracted to the trap were captured alive by the suction device. Gravid traps are particularly valuable for virus surveillance because they selectively capture females that have completed at least one gonotorphic cycle and are thus more likely to be infected with a pathogen acquired through blood feeding. The selective pooling for virus isolation of a female cohort collected in gravid traps increases the probability of detecting and identifying mosquito-borne viruses that are being transmitted close to the area sampled by the trap.

Ovitraps contain oviposition attractants and are designed to collect either gravid mosquitoes or mosquito eggs, providing an indication of the relative abundance and species composition of mosquitoes in the area sampled by the trap. Ovitraps have been used to sample *Culex* [84] and *Aedes* [85] eggs as well as *Culex* and *Aedes* adults [86,87]. For example, an ovitrap with the inside wall treated with a polybutylene adhesive was as effective as a standard ovitrap at detecting *Ae. aegypti* while also capturing *Ochlerotatus notoscriptus* (Skuse) and *Cx. quinquefasciatus* in Cairns, Australia [88].

### 3.2. Oviposition Behavior and Vector Control

The successful use of gravid and ovitraps for vector surveillance lead to the suggestion that these devices may be effective for vector control. This idea was particularly exciting because theses traps selectively sample older cohorts of the mosquito population that may be actively involved in disease transmission. Attractant ovitraps have been used in an attract-and-kill vector control program against *Cx. quinquefasciatus* in Recife, Brazil where traps containing the oviposition attractant skatol or a fermented grass infusion were treated with *Bacillus thuringiensis* variety *israelensis* (Bti). Traps baited with attractants and treated with Bti lured significantly more gravid mosquitoes that oviposited in the traps than did traps baited with water alone [7].

Attract-and-kill strategies have also been designed for the control of *Ae. aegypti* in areas where they transmit dengue viruses. Several generations of a disposable, long-lasting autocidal gravid ovitrap treated with a non-setting, polybutylene adhesive designed to ensnare gravid females as they enter the trap have been developed and successfully tested in the laboratory and under semi-field and field conditions [89]. Lethal ovitraps used in attract-and-kill vector control programs have been successful in the control of *Ae. aegypti* in Australia where the public acceptance of this trapping program was high [90]. By saturating a test area with lethal ovitraps it was shown that the attract-and-kill control strategy can significantly impact *Ae. aegypti* populations [91]. Laboratory, semi-field, and field experiments in north Queensland, Australia with lethal ovitraps containing the pyrethroid insecticide bifenthrin indicated that bifenthrin-treated ovitraps were repellent to gravid *Ae. aegypti*, but that lethal contact occurred without oviposition, suggesting that these traps have potential for additional levels of *Ae. aegypti* control in integrated vector control programs [92].

## 4. Conclusions

Oviposition is the bridge between mosquito generations, and its importance to female mosquitoes is illustrated by the willingness of dying gravid females to oviposit in virtually any source of water, including wet paper towels, on the remote chance that eggs will hatch and larvae develop. Oviposition, like blood feeding, evolved along several lines to include species that are specialists, inflexible in their selection of an oviposition site, to those that are generalists, ovipositing in virtually any aquatic habitat. Specialists like *Deinocerites cancer* rely on the very specific habitat of partially flooded crab holes for oviposition (Figure 3). When crab holes disappear, so do the *Deinocerites* that dwell in them (Personal Observation). Generalists, such as *Culex nigripalpus*, oviposit in just about any water source. To be sure, Cx. nigripalpus have preferred oviposition sites and when these preferred sites flood the local abundance of this mosquito species increases dramatically. In addition, mosquitoes have evolved egg-laying strategies that include deposition of RH eggs, DH eggs, and combinations of both. Eggs are deposited individually, in rafts, in clusters, on the water, near the water, and on dry land. Oviposition attractants work over long, medium, short, and contact distances and mosquitoes have evolved a variety of mechanisms to accurately evaluate the quality and potential longevity of the sites into which their eggs are deposited. Which strategy is the most effective? A behavioral flexibility for determining where and when eggs are laid is probably the safest strategy. For example, the timing of *Cx. tarsalis* oviposition is controlled by physical, chemical, and behavioral cues. This species is flexible enough in its behavior so that in the absence of one set of cues (i.e., visual signals), oviposition continues to completion when other cues (i.e., heat, moisture, and olfaction) take over [93]. As with other insect species, habitat availability is a key factor in determining where a mosquito will oviposit and how successful it will be in terms of its overall abundance. Populations of salt marsh mosquitoes (*Ae. taeniorhynchus* and *Ae. sollicitans*) along the east coast of Florida dropped dramatically during the 1970s and 1980s. This was due mainly to the implementation of marsh impoundment techniques [36] that modified thousands of acres of the preferred oviposition habitat of these mosquitoes rendering the modified habitat unacceptable for mosquito egg laying. On the other hand, *Ae. vexans* in southeast Florida experienced a population increase beginning in the early 1970s, due mainly to the introduction of citrus farming techniques that favored the creation of oviposition habitats preferred by this species [94]. Rare mosquito species, such as *Ae. fulvus pallens* Ross in south Florida, have nearly disappeared as their preferred woodland oviposition sites have been cleared, paved, and developed (Personal Observation). Mosquitoes will always be with us. Some species will be flexible enough to adapt to blood feed and oviposit in habitats associated with our homes, cities, industrial centers, and agricultural land. Other species will be unable to adjust to local habitat loss and will go extinct. Most mosquito species remain nothing more than a nuisance to humans. Others are dangerous disease vectors. Vector control efforts will remain important wherever mosquitoes have water in which to oviposit. A thorough understanding of mosquito oviposition has already helped in the development of novel mosquito surveillance and control strategies to accurately track populations and to better control these important and dangerous insects.

## Figures and Tables

**Table 1:** Mosquito oviposition: examples of egg laying behavior [1,8].

Method of Oviposition	Tribe	Genera	Species
**In flight emission of single eggs to the oviposition site**		*Anopheles* Meigen	*Anopheles atroparvus* Van Thiel
			*Anopheles gambiae* Giles
	Sabethini	*Sabethes* Robineau-Desvoidy	*Sabethes chloropterus* (Von Humboldt)
	Sabethini	*Tripteroides* Giles	*Tripteroides aranoides* (Theobald)
	Sabethini	*Topomyia* Leicester	*Topomyia yanbarensis* Miyagi
	Sabethini	*Wyeomyia* Theobald	*Wyeomyia vanduzeei* Dyar and Knab
	Toxorhynchitini	*Toxorhynchites* Theobald	*Toxorhynchites amboinensis* (Doleschall)
**Desiccation-resistant eggs laid singly on moist surfaces near standing water**	Aedini	*Aedes* Meigen	*Aedes aegypti* (Linnaeus)
	Aedini	*Aedes* Meigen	*Aedes triseriatus* (Say)
	Aedini	*Armigeres* Theobald	*Armigeres subalbatus* (Coquillett)
	Culicini	*Deinocerites* Theobald	*Deinocerites cancer* Theobald
	Aedini	*Haemagogus* Williston	*Haemagogus equinus* Theobald
	Aedini	*Opifex* Hutton	*Opifex fuscus* Hutton
	Aedini	*Psorophora* Robineau-Desvoidy	*Psorophora ferox* (Von Humboldt)
**Egg rafts laid on the water surface**	Aedini	*Armigeres* Theobald	*Armigeres digitatus* (Edwards)
	Culicini	*Culex* Linnaeus	*Culex nigripalpus* Theobald
	Culisetini	*Culiseta* Felt	*Culiseta melanura* (Coquillett)
	Mansoniini	*Coquillettidia* Dyar	*Coquillettidia aurites* (Theobald)
	Uranotaeniini	*Uranotaenia* Lynch Arribalzaga	*Uranotaenia sapphirina* (Osten Sacken)
**Desiccation-resistant egg rafts laid on moist surfaces near standing water**	Culisetini	*Cuicella* Felt	*Cuicella morsitans* (Theobald)
**Egg clusters cemented to surfaces of aquatic plants**	Ficalbiini	*Ficalbia* Theobald	*Ficalbia minima* (Theobald)
	Mansoniini	*Mansonia* Blanchard	*Mansonia titillans* (Walker)
**Female guards a cluster of eggs (egg brooding)**	Aedini	*Armigeres* Theobald	*Armigeres flavus* (Leicester)
	Sabethini	*Trichoprosopon* Theobald	*Trichoprosopon digitatum* (Rondani)

## References

[1] Clements A.N. (1996). The Biology of Mosquitoes, Development, Nutrition and Reproduction.

[2] Bidlingmayer W.L. (1974). The influence of environmental factors and physiological stage on flight patterns of mosquitos taken in the vehicle aspirator and truck, suction, bait and New Jersey light traps. J. Med. Entomol..

[3] Bidlingmayer W.L. (1975). Mosquito flight paths in relation to the environment. Effect of vertical and horizontal visual barriers. Ann. Entomol. Soc. Am..

[4] Bentley M., Day J. (1989). Chemical ecology and behavioral aspects of mosquito oviposition. Ann. Rev. Entomol..

[5] Day J., Curtis G.A. (1994). When it rains they soar—And that makes *Culex nigripalpus* a dangerous mosquito. Am. Entomol..

[6] Reiter P. (1983). A portable battery-powered trap for collecting gravid *Culex* mosquitoes. Mosq. News.

[7] Ritchie S.A., Long S., Hart A., Webb C.E., Russell R.C. (2003). An adulticidal sticky ovitrap for sampling container-breeding mosquitoes. J. Am. Mosq. Control Assoc..

[8] Mosquito Taxonomic Inventory. http://mosquito-taxonomic-inventory.info/.

[9] Bidlingmayer W.L., Hem D.G. (1979). Mosquito (Diptera: Culicidae) flight behaviour near conspicuous objects. Bull. Entomol. Res..

[10] Gillies M. (1972). Some aspects of mosquito behavior in relation to the transmission of parasites. Zool. J. Linn. Soc..

[11] Macan T.T. (1961). Factors that limit the range of freshwater animals. Biol. Rev..

[12] Lounibos L.P. (1978). Mosquito breeding and oviposition stimulant in fruit husks. Ecol. Entomol..

[13] Dethier V.G., Browne L.B., Smith C.N. (1960). The designation of chemicals in terms of the responses they elicit from insects. J. Econ. Entomol..

[14] Lowenberger C.A., Rau M.E. (1994). Selective oviposition by *Aedes aegypti* (Diptera: Culicidae) in response to a larval parasite, *Plagiorchis elegans* (Trematoda: Plagiorchiidae). Environ. Entomol..

[15] Kennedy J.S. (1978). The concepts of olfactory arrestment and attraction. Physiol. Entomol..

[16] Bidlingmayer W.L., Day J.F., Evans D.G. (1995). Effect of wind velocity on suction trap catches of some Florida mosquitoes. J. Am. Mosq. Control Assoc..

[17] Dow R.P., Gerrish G.M. (1970). Day-to-day change in relative humidity and the activity of *Culex nigripalpus* (Diptera: Culicidae). Ann. Entomol. Soc. Am..

[18] Bidlingmayer W.L., Franklin B.P., Jennings A.M., Cody E.F. (1974). Mosquito flight paths in relation to the environment. Influence of blood meals, ovarian stage and parity. Ann. Entomol. Soc. Am..

[19] Birley M.H., Charlwood J.D. (1989). The effects of moonlight and other factors on the oviposition cycle of malaria vectors in Madang, Papua new Guinea. Ann. Trop. Med. Parasitol..

[20] O’Gower A.K. (1963). Environmental stimuli and the oviposition behavior of *Aedes aegypti* var. *queenslandensis* Theobald (Diptera: Culicidae). Anim. Behav..

[21] Belton P. (1967). Effect of illumination and pool brightness on oviposition by *Culex restuans* (Theo) in the field. Mosq. News.

[22] Kennedy J.S. (1942). On water-finding and oviposition by captive mosquitoes. Bull. Entomol. Res..

[23] Gibson G. (1995). A behavioural test of the sensitivity of a nocturnal mosquito, *Anopheles gambiae*, to dim white, red, and infra-red light. Physiol. Entomol..

[24] Wellington W.G. (1974). Change in mosquito flight associated with natural changes in polarized light. Can. Entomol..

[25] Silberglied R.E. (1979). Communication in the ultra-violet. Ann. Rev. Ecol. Syst..

[26] Focks D.A., Sackett S.R., Dame D.A., Bailey D.L. (1983). Ability of *Toxorhynchites amboinensis* (Doleschall) (Diptera: Culicidae) to locate and oviposit in artificial containers in an urban environment. Environ. Entomol..

[27] Choo Y.-M., Buss G.K., Tan K., Leal W.S. (2015). Multitasking roles of mosquito labrum in oviposition and blood feeding. Front. Physiol..

[28] Thavara U., Tawatsin A., Chompoosri J. (2004). Evaluation of attractants and egg-laying substrate preference for oviposition by *Aedes albopictus* (Diptera: Culicidae). J. Vector Ecol..

[29] Beehler J., Lohr S., DeFoliart G. (1992). Factors influencing oviposition in *Aedes triseriatus* (Diptera: Culicidae). Great Lakes Entomol..

[30] McCrae A.W.R. (1984). Oviposition by African malaria vector mosquitoes. II. Effects of site tone, water type and conspecific immatures on target selection by freshwater *Anopheles gambiae* Gilles, *sensu lato*. Ann. Trop. Med. Parasitol..

[31] Knight K.L., Baker T.E. (1962). The role of the substrate moisture content in the selection of oviposition sites by *Aedes taeniorhynchus* (Wicd.) and *A. sollicitans* (Walk.). Mosq. News.

[32] Jackson M.L. (1958). Soil Chemical Analysis.

[33] Olson J.K., Meek C.L. (1977). Soil moisture conditions that are most attractive to ovipositing females of *Psorophora columbiae* in Texas ricefields. Mosq. News.

[34] Meek C.L., Williams D.C. (1986). Diel periodicity of oviposition and soil moisture preference of *Psorophora columbiae*. J. Entomol. Sci..

[35] Petranka J.W., Fakhoury K. (1991). Evidence of a chemically-mediated avoidance response of ovipositing insects to bluegills and green frog tadpoles. Copeia.

[36] Ault S.K. (1994). Environmental management: A re-emerging vector control strategy. Am. J. Trop. Med. Hyg..

[37] Isoe J., Beehler J.W., Millar J.G., Mulla M.S. (1995). Oviposition responses of *Culex tarsalis* and *Culex quinquefasciatus* to aged Bermuda grass infusions. J. Am. Mosq. Control Assoc..

[38] Russell R.C. (1999). Constructed wetlands and mosquitoes: Health hazards and management options—An Australian perspective. Ecol. Eng..

[39] Burkett-Cadena N.D., Mullen G.R. (2007). Field comparison of Bermuda-hay infusion to infusions of emergent aquatic vegetation for collecting female mosquitoes. J. Am. Mosq. Control Assoc..

[40] Du Y.-J., Millar J.G. (1999). Electroantennogram and oviposition bioassay responses of *Culex quinquefasciatus* and *Culex tarsalis* (Diptera: Culicidae) to chemicals in odors from Bermuda grass infusions. J. Med. Entomol..

[41] Trexler J.D., Apperson C.S., Schal C. (1998). Laboratory and field evaluations of oviposition responses of *Aedes albopictus* and *Aedes triseriatus* (Diptera: Culicidae) to oak leaf infusions. J. Med. Entomol..

[42] Dawson G.W., Laurence B.R., Pickett J.A., Pile M.M., Wadhams L.J. (1989). A note on the mosquito oviposition pheromone. Pestic. Sci..

[43] Iltis W.G., Zweig G. (1962). Surfactant in apical drop of eggs of some culicine mosquitoes. Ann. Entomol. Soc. Am..

[44] Van Pletezen R., van der Linde T.C.de.K. (1981). Studies on the biology of *Culiseta longiareolata* (Macquart) (Diptera: Culicidae). Bull. Entomol. Res..

[45] Allan S., Kline D. (1995). Evaluation of organic infusions and synthetic compounds mediating oviposition in *Aedes albopictus* and *Aedes aegypti* (Diptera: Culicidae). J. Chem. Ecol..

[46] Chadee D.D. (1993). Oviposition response of *Aedes aegypti* (L.) to the presence of conspecific eggs in the field in Trinidad, W.I.. J. Fla. Mosq. Control Assoc..

[47] Edgerly J.S., McFarland M., Morgan P., Livdahl T. (1998). A seasonal shift in egg-laying behaviour in response to cues of future competition in a treehole mosquito. J. Anim. Ecol..

[48] Benzon G.L., Apperson C.S. (1988). Reexamination of chemically mediated oviposition behavior in *Aedes aegypti* (L.) (Diptera: Culicidae). J. Med. Entomol..

[49] Kalpage D.S.P., Brust R.A. (1973). Oviposition attractant produced by immature *Aedes atropalpus*. Environ. Entomol..

[50] Trimble R.M., Wellington W.G. (1980). Oviposition stimulant associated with fourth-instar larvae of *Aedes togoi* (Diptera: Culicidae). J. Med. Entomol..

[51] Maire A. (1985). Effect of axenic larvae on the oviposition site selection by *Aedes atropalpus*. J. Am. Mosq. Control Assoc..

[52] Hudson A., McLintock J. (1967). A chemical factor that stimulates oviposition by *Culex tarsalis* Coquillett (Diptera, Culicidae). Anim. Behav..

[53] Osgood C.E. (1971). An oviposition pheromone associated with the egg rafts of *Culex tarsalis*. J. Econ. Entomol..

[54] Roberts D.R., Hsi B.P. (1977). A method of evaluating ovipositional attractants of *Aedes aegypti* (Diptera: Culicidae), with preliminary results. J. Med. Entomol..

[55] Hwang Y.S., Schultz G.W., Mulla M.S. (1984). Structure-activity relationship of unsaturated fatty acids as mosquito ovipositional repellents. J. Chem. Ecol..

[56] Klocke J.A., Darlington M.V., Balandrin M.F. (1987). Biologically-active constituents of North-American plants. Part III. 1,8-Cineole (Eucalyptol), a mosquito feeding and ovipositional repellent from volatile oil of *Hemizonia fitchii* (Asteraceae). J. Chem. Ecol..

[57] Pappas L.G., Pappas C.D. (1983). Laboratory studies on the significance of NaCl as an oviposition deterrent in *Culiseta inornata*. Mosq. News.

[58] Zahiri N., Rau M.E. (1998). Oviposition attraction and repellency of *Aedes aegypti* (Diptera: Culicidae) to waters from conspecific larvae subjected to crowding, confinement, starvation or infection. J. Med. Entomol..

[59] Ritchie S.A., Laidlaw-Bell C. (1994). Do fish repel oviposition by *Aedes taeniorhynchus?*. J. Am. Mosq. Control Assoc..

[60] Curtis G.A. (1985). Habitat selection strategies of mosquitoes inhabiting citrus irrigation furrows. J. Am. Mosq. Control Assoc..

[61] Chaves L.F., Kitron U.D. (2011). Weather variability impacts on oviposition dynamics of the southern house mosquito it intermediate time scales. Bull. Entomol. Res..

[62] Andersson I.H., Jaenson T.G.T. (1987). Nectar feeding by mosquitoes in Sweden with special reference to *Culex pipiens* and *Cx. torrentium*. Med. Vet. Entomol..

[63] Klowden M.J., Dutro S.M. (1990). Effects of carbohydrate ingestion on the pre-oviposition behavior of the mosquito *Aedes aegypti* (L.). Bull. Soc. Vector Ecol..

[64] Shroyer D.A., Sanders D.P. (1977). The influence of carbohydrate-feeding and insemination on oviposition of and Indiana strain of *Aedes vexans* (Diptera: Culicidae). J. Med. Entomol..

[65] Weber R.G., Tipping C. (1990). Drinking as a pre-oviposition behavior of wild *Culex pipiens* (Diptera: Culicidae). Entomol. News.

[66] Weber R.G., Tipping C. (1993). Preoviposition drinking by *Culex restuans* (Diptera: Culicidae). J. Insect Behav..

[67] Gillett J.D. (1962). Contributions to the oviposition cycle by individual mosquitoes in a population. J. Insect Physiol..

[68] Gillett J.D., Corbet P.S., Haddow A.J. (1959). Observations on the oviposition-cycle of *Aedes* (*Stegomyia*) *aegypti* (Linnaeus) III. Ann. Trop. Med. Parasitol..

[69] Gillett J.D., Corbet P.S., Haddow A.J. (1961). Observations on the oviposition-cycle of *Aedes* (*Stegomyia*) *aegypti* (Linnaeus). VI. Ann. Trop. Med. Parasitol..

[70] Gillett J.D., Haddow A.J., Corbet P.S. (1959). Observations on the oviposition-cycle of *Aedes* (*Stegomyia*) *aegypti* (Linnaeus). II. Ann. Trop. Med. Parasitol..

[71] Haddow A.J., Gillett J.D., Corbet P.S. (1961). Observations on the oviposition-cycle of *Aedes* (*Stegomyia*) *aegypti* (Linnaeus). V. Ann. Trop. Med. Parasitol..

[72] Lounibos L.P., Linley J.R. (1987). A quantitative analysis of underwater oviposition by the mosquito *Mansonia titillans*. Physiol. Entomol..

[73] Russell P.F., Rao T.R. (1942). On relation of mechanical obstruction and shade to oviposition of *Anopheles culicifacies*. J. Exp. Zool..

[74] Galindo P. (1958). Bionomics of *Sabethes chloropterus* Humboldt, a vector of sylvan yellow fever in Middle America. Am. J. Trop. Med. Hyg..

[75] Linley J.R. (1987). Aerial oviposition flight of *Toxorhynchites amboinensis* (Diptera: Culicidae). J. Med. Entomol..

[76] Beament J. (1989). John Hull Grundy lecture. Eggs—The neglected insects. J. R. Army Med. Corps.

[77] Apostol B.L., Black W.C., Reiter P., Miller B.R. (1994). Use of randomly amplified polymorphic DNA amplified by polymerase chain reaction markers to estimate the number of *Aedes aegypti* families at oviposition sites in San Juan, Puerto Rico. Am. J. Trop. Med. Hyg..

[78] Irving-Bell R.J., Inyang E.N., Tamu G. (1991). Survival of *Aedes vittatus* (Diptera: Culicidae) eggs in hot dry rockpools. Trop. Med. Parasitol..

[79] Beier J.C., Copeland R., Oyaro C., Masinya A., Odago W.O., Oduor S., Koech D.K., Roberts C.R. (1990). *Anopheles gambiae* complex egg-stage survival in dry soil from larval development sites in western Kenya. J. Am. Mosq. Control Assoc..

[80] Lounibos L.P., Dewald L.B. (1989). Oviposition site selection by *Mansonia* mosquitoes on water lettuce. Ecol. Entomol..

[81] Okazawa T., Miyagi I., Toma T., Ramalingam S., Chang M.S. (1991). Egg morphology and observations on the laboratory biology of *Armigeres* (*Leicesteria*) *digitalus* (Diptera: Culicidae) from Sarawak. J. Med. Entomol..

[82] Lounibos L.P., Machado-Allison C.E. (1983). Oviposition an egg brooding by the mosquito *Trichoprosopon digitatum* in cacao husks. Ecol. Entomol..

[83] Lounibos L.P., Machado-Allison C.E. (1987). Female brooding protects mosquito eggs from rainfall. Biotropica.

[84] Barbosa R.M.R., Regis L.N. (2011). Monitoring temporal fluctuations of *Culex quinquefasciatus* using oviposition traps containing attractants and larvicide in a urban environment in Recife, Brazil. Mem. Inst. Oswaldo Cruz.

[85] Chadee D.D., Corbet P.S. (1987). Seasonal incidence and diel patterns of oviposition in the field of the mosquito *Aedes aegypti* (Diptera: Culicidae) in the field in Trinidad, W.I.: A preliminary study. Ann. Trop. Med. Parasitol..

[86] Zhang L.-Y., Lei C.L. (2008). Evaluation of sticky ovitraps for the surveillance of *Aedes* (*Stegomyia*) *albopictus* (Skuse) and the screening of oviposition attractants from organic infusions. Ann. Trop. Med. Parasitol..

[87] Eiras A.E., Buhagiar T.S., Richie S.A. (2014). Development of the gravid *Aedes* trap for the capture of adult female container-exploiting mosquitoes (Diptera: Culicidae). J. Med. Entomol..

[88] Barbosa R.M.R., Regis L., Vasconcelos R., Leal W.S. (2010). *Culex* mosquitoes (Diptera: Culicidae) egg laying in traps loaded with *Bacillus thuringiensis* variety *israelensis* and baited with skatole. J. Med. Entomol..

[89] Mackay A.J., Amador M., Barrera R. (2013). An improved autocidal gravid ovitrap for the control and surveillance of *Aedes aegypti*. Parasites Vectors.

[90] Ritchie S.A., Rapley L.P., Williams C., Johnson P.H., Larkman M., Silcock R.M., Long S.A., Russell R.C. (2009). A lethal ovitrap-based mass trapping scheme for dengue control in Australia: I. Public acceptability and performance of lethal ovitraps. Med. Vet. Entomol..

[91] Rapley L.P., Johnson P.H., Williams C.R., Silcock R.M., Larkman M., Long S.A., Russell R.C., Ritchie S.A. (2009). A lethal ovitrap-based mass trapping scheme for dengue control in Australia: II. Impact on populations of the mosquito *Aedes aegypti*. Med. Vet. Entomol..

[92] Williams C.R., Ritchie S.A., Long S.A., Dennison N., Russell R.C. (2007). Impact of bifenthrin-treated lethal ovitrap on *Aedes aegypti* oviposition and mortality in North Queensland, Australia. J. Med. Entomol..

[93] Isoe J., Millar J.G. (1996). Water deprivation and light affect responses of *Culex tarsalis* (Diptera: Culicidae) to oviposition site cues. Environ. Entomol..

[94] Curtis G.A., Frank J.H. (1981). Establishment of *Aedes vexans* in citrus groves in southeastern Florida. Environ. Entomol..

